# The Societal Readiness Thinking Tool: A Practical Resource for Maturing the Societal Readiness of Research Projects

**DOI:** 10.1007/s11948-021-00360-3

**Published:** 2022-01-27

**Authors:** Michael J. Bernstein, Mathias Wullum Nielsen, Emil Alnor, André Brasil, Astrid Lykke Birkving, Tung Tung Chan, Erich Griessler, Stefan de Jong, Wouter van de Klippe, Ingeborg Meijer, Emad Yaghmaei, Peter Busch Nicolaisen, Mika Nieminen, Peter Novitzky, Niels Mejlgaard

**Affiliations:** 1 AIT, Austrian Institute of Technology, GmbH, Vienna, Austria; 2 Department of Sociology, University of Copenhagen, København, Denmark; 3 Danish Centre for Studies in Research and Research Policy, Aarhus University, Aarhus C, Denmark; 4 Centre for Science and Technology Studies, Leiden University, Leiden, The Netherlands; 5 Institute for Advanced Studies, Vienna, Austria; 6 Department of Organization Studies, Tilburg University, Tilburg, The Netherlands; 7 Knowledge Lab, Department of Sociology, Division of the Social Sciences, The University of Chicago, Chicago, IL USA; 8 Centre for Research on Evaluation, Science and Technology and the DST-NRF Centre for Excellence in Scientometrics and Science, Technology and Innovation Policy, Faculty of Arts and Social Sciences, Stellenbosch University, Stellenbosch, South Africa; 9 Faculty of Technology, Policy and Management, Delft University of Technology, Delft, The Netherlands; 10 VTT Technical Research Centre of Finland, Espoo, Finland; 11 Wageningen University & Research, Wageningen, The Netherlands; 12 School for the Future of Innovation in Society, Arizona State University, Tempe, Arizona USA

**Keywords:** Innovation, TRL, Technology Readiness Levels, RRI, Responsible research and innovation, Research management, Societal Readiness, Thinking Tool

## Abstract

In this paper, we introduce the Societal Readiness (SR) Thinking Tool to aid researchers and innovators in developing research projects with greater responsiveness to societal values, needs, and expectations. The need for societally-focused approaches to research and innovation—complementary to Technology Readiness (TR) frameworks—is presented. Insights from responsible research and innovation (RRI) concepts and practice, organized across critical stages of project-life cycles are discussed with reference to the development of the SR Thinking Tool. The tool is designed to complement not only shortfalls in TR approaches, but also improve upon other efforts to integrate RRI, sustainability, and design thinking in research and innovation cycles. Operationalization and early-stage user tests of the Tool are reported, along with discussion of potential future iterations and applications.

**Supplementary Information:**

The online version contains supplementary material available at 10.1007/s11948-021-00360-3.

## Introduction

Scientific and technological research and innovation seem bound in a challenge of advancing human achievements on and beyond Earth, without also succumbing to unintended, undesirable consequences for all forms of life on the planet (Merton, 1936). Too often, the very innovations to which people turn for solutions generate new, cascading sets of second, third, and fourth order undesirable effects (Westley et al., 2011). In parallel, government, private sector, and international entities increasingly call for “impacts,” whether as commercialization or innovation to address sustainability challenges, (Smith and Bandola-Gill, 2020; Adams et al., 2016; OECD, 2011), with little attention to the role of science and technology in perpetuating inequalities (Bozeman et al., 2011; Woodhouse & Sarewitz, 2007). Linear approaches like the Technology Readiness Level (TRL) system (Mankins, 1995), contribute to a drumbeat of technology development for impact with limited consideration of institutional or societal “readiness” (Webster & Gardner, 2019a, 2019b). The concept of responsible research and innovation (RRI) has been intentionally cultivated across Europe and beyond to develop greater responsiveness of research and innovation (R&I) to societal values and ethical concerns (von Schomberg, 2011). However, means of supporting RRI adoption, while diverse, are often limited to singular dimensions, disconnected from, or unintegrated across R&I processes and practices (Schuijff & Dijkstra, 2020; Shelley-Egan et al., 2018).

In this article, we present a Societal Readiness (SR) Thinking Tool to support scientist and engineers in anticipating and reflecting on social and ethical dimensions of research and innovation processes. We do so by collecting, structuring, and curating a range of resources to support researchers to, depending on their preferred manner, scaffold or spark thinking about the societal implications of their work. Our emphasis on a broad notion of “societal readiness” complements and seeks to “open up” commonly linear “technology readiness” approaches to scaffolding innovation activities (Stirling, 2008). Through such opening, our hope is to make a modest contribution to loosening the grip of cycles of cascading, undesirable, inequitable impacts of innovations in society.

### From Technology Readiness to Societal Readiness

One of the more popular approaches to technology development and advancement—regardless of relevance to social or ethical dimensions—is the idea of thinking about ‘readiness.’ During the 1980s and 1990s, the National Aeronautics and Space Administration (NASA) developed the method of Technology Readiness Level to quantify the maturity of a given technology (Mankins, 1995). Initially developed as an inter-organizational method to enable technology-push projects in the Space and Weapons industry, TRL offers an assessment framework for determining technical and economic costs, prospective economic value, and possible risks associated with a technology (Héder, 2017; Webster & Gardner, 2019a, 2019b). Moreover, as a standardized management framework, TRL is used to facilitate cooperation among a diverse set of stakeholders, including designers, engineers, funding agencies, and regulators (Webster & Gardner, 2019a, 2019b). TRL spans nine levels, from the initial scientific validation of an idea to its full commercial application, with each level indicating an improvement in technological maturation. Each level represents a “risk-gate” for a technology to pass through and demonstrate “readiness,” within a bounded operational environment (Webster & Gardner, 2019a, 2019b).

The TRL method is widely embraced by state agencies in large-scale and high-risk settings (e.g., the US Federal Aviation Authority, the US department of Energy and the UK Nuclear Decommissioning Authority), and by companies in aeronautical, automotive, nanotechnology and other industries (Dreyer et al., 2017; Webster & Gardner, 2019a, 2019b). Innovation and funding agencies such as the UK Engineering and Physical Sciences Research Council and the European Commission have adopted the TRL methodology to help guide scientists and innovators through early stages of the research and development process (i.e., technology readiness level 1–3, where basic principles are identified, technological concepts are formulated, and experiments are conducted) (Stahl, 2013; Owen & Goldberg, 2010; EPSRC, 2019). Rybicka et al. (2016), Galdysz and Kluczek (2017), and Reißmann et al. (2018), for example, each referenced TRL as a foundational approach to assessing technology performance improvement and commercialization potential.

Arguing that successful technology development requires not only high levels of technical sophistication of a separate technology but also integration within systems of technologies, Sauser et al. (2006) advanced the complementary notion of Integration Readiness Level (IRL), which assesses the degree to which two technologies can successfully be integrated, alongside the notion of System Readiness Levels (SysRL), which focuses on the viability of systems of interdependent technologies. Acknowledging that technologies also need humans to operate them, several additional scales have subsequently been developed with the aim of assessing the human aspects of operating technologies. These include Human Readiness Levels (HRL) (Phillips, 2010) Human Factors Readiness Levels (HFRL) (Giudice et al., 2015; Hale et al., 2011), Human Capability Level (HCL) and Human Integration Readiness Level (HIRL) (Miller et al., 2016).

These attempts to capture ‘readiness’ are similar in how they employ taxonomies, implicitly presuppose linear development trajectories towards higher levels of ‘readiness’, and focus on technical adequacy. They are concerned with whether technologies *can* perform the functions they are expected to but not whether such functions are socially desirable. In contrast, a different set of taxonomies aspire to describe the level to which society may *want* a technology. Paun (2012) offered the notion of Demand Readiness Level (DRL) and Hjort and Brem (2016) introduced Market Readiness Level (MRL), taking a macro pull perspective on technology development by looking at the (collective) readiness of society to apply a given technology. Approaching the issue from a micro pull perspective, Parasuraman (2000) and Parasuraman and Colby (2015) proposed the notion of Technology Readiness Index (TRI), which considers the readiness of an individual to embrace a given technology. Such approaches often still adhere to a linearity in how society is conceptualized as an external entity waiting for a technology to apply or consume.

Examining these approaches to Technology Readiness reveals the way in which responsibility for aligning innovation products and processes with long-term societal values, ethical concerns, or broader interests, is compartmentalized. Researchers and innovators are primarily responsible for considering whether a technology ‘will work’—often driven by industry or customer requirements (Tomaschek et al., 2016). Citizens and societal stakeholders, often in separate activities, are primarily expected to deal with the issue of whether a technology ‘is wanted.’ Webster and Gardner (2019a, 2019b) argued that TRL methods, due to a primary focus on configuring technological systems-components, tools and devices, did not make explicit the social factors shaping the maturation of new technologies. Moreover, where social factors are considered, Iatridis and Schroeder (2016) argued that TRL and related methods for technology assessment tend to be more occupied with contractual and legal risks associated with a technology rather than broader ethical considerations about social acceptability. While TRL methods ask, “will it work?”, and demand-concerned taxonomies ask, “will anyone want it?”, neither ask, “will it acceptably address broader, long-term societal concerns?”.

### Addressing Gaps in Technology Readiness Through Responsible Research and Innovation

Ensuring research and innovation integrate broader societal and ethical concerns, von Schomberg (2014) argued, requires careful attention to the social processes through which scientific knowledge and innovations are shaped. Further, he argued that the concept of responsible research and innovation (RRI) can help steer R&I processes toward shared societal objectives such as sustainable economic growth, social justice, gender equality, and protection of human health and the environment (von Schomberg, 2014, 34–36). Concurrently, van de Poel et al. (2020) observed three ways in which RRI tools and assessments offer added value to innovation processes. The first proposition related to opening conversations to a broader array of values and issues. The second revolved around engaging external perspectives in conversations about these values and issues. The third related to the utility of conducting such expansive and integrative reflection early in the process of project ideation and development. The concept of RRI has gained prominence in the academic literature, observable in the rapid increase in the number of publications addressing RRI since the late 2000s (Genus and Iskandarova, 2018), RRI practices between 2005 and 2015 (Schuijff & Dijkstra, 2020), and investment by the European Commission of approximately €1.88 billion to advance RRI across topics spanning quantum computing and energy technology research to investigations into the human brain and artificial intelligence (Novitzky et al., 2020).

In a way that particularly suits a complementary approach to TRLs, “responsibilities” covered by RRI extend beyond conventional scientific notions such as research integrity, ethical reviews, or codes of conduct to also hold in regard an anticipatory concern for unintended, undesirable consequences (Owen et al., 2012; Stilgoe et al., 2013). The aim of RRI approaches is to support researchers and innovators in more actively anticipating, engaging, and acting to ensure the social acceptability of their work in the short- and long-term. The European Commission (EC) consistently highlights the potential of RRI for helping advance R&I to tackle grand societal challenges related to health, food security, clean energy, transport, climate, social inclusion, and privacy rights (Geoghegan-Quinn, 2012).

The most common definition of RRI and the one adopted by the Commission was coined by former EC official René von Schomberg. According to von Schomberg (2011, p. 9) RRI is:

…a transparent, interactive process by which societal actors and innovators become mutually responsive to each other with a view on the (ethical) acceptability, sustainability and societal desirability of the innovation process and its marketable products (in order to allow a proper embedding of scientific and technological advances in our society).

RRI, like technology development, is represented as a process rather than an outcome (Burget et al., 2017) and is one still actively “in the making” (Owen et al., 2021). Amidst sustained efforts, translating RRI into practice has proven challenging (Blok & Lemmens, 2015; Burget et al., 2017; Felt et al., 2018). Schuijff and Dijkstra (2020), in a review of academic publications featuring RRI practices between 2005 and 2015, observed limitations in addressing reflective and anticipatory concerns throughout R&I processes (e.g., as opposed to only at beginning or midstream); or in focusing on a single versus the suite of RRI dimensions. Despite its current limitations in implementation, value propositions for attempting RRI remain. Stahl et al. (2019), for example, focusing on Information and Communication Technology industry activities and RRI, identified the usefulness of RRI in particular for instances where societal concerns may be in company “blind-spots”, difficult to map to “organizational objectives”, or “not formally regulated” (Stahl et al., 2019).

### Developing the Societal Readiness Thinking Tool

We introduce the Societal Readiness (SR) Thinking Tool^1^ as a practical resource for scientists and engineers who wish to integrate broader social and ethical dimensions of responsibility into their practices. We chose to emphasize “thinking” as a central, iterative activity unfolding across phases of research and innovation projects. The design intent is for the tool to “spark thinking” at any or all aspects of a project lifecycle. Questions posed by the tool challenge users to “think across” a range of societal issues; “think through” responses considering planned or existing projects; and “think with” colleagues and stakeholders on how to respond with modifications in practice.

The SR Thinking Tool—as distinct from existing RRI resources—organizes and offers concrete questions across all stages of research and innovation activities, as well as the full spectrum of RRI concerns. Architected in this manner, the SR Thinking Tool directly responds to critiques of how RRI implementation lacks concreteness, is often divorced from practice (Shelley-Egan et al., 2018) or disjointed relative to R&I processes or across RRI concerns (Schuijff & Dijkstra, 2020). Developing a thinking tool to cover such a broad and deep topic required a flexible approach; one acknowledging the versatile and pluralistic nature of project-based research. Such a resource needed to be detailed enough to stimulate appropriate reflection and action, yet general enough to be applicable in different research contexts (Owen, 2014). To ensure such flexibility, we conceived of the thinking tool as a dynamic instrument, open to continuous context- and field-specific adjustments and refinements.

The SR Thinking Tool departs from a variety of resources developed by RRI researchers and other scholars interested in advancing broader societal dimensions of technology and innovation (See Supplementary Material, 1.3S). Distinct from IMAGINE RRI (Felt et al., 2018), RRI Tools (2014, 2018), and various RRI applications for business use (Blok & Lemmens, 2015; Van de Poel et al., 2017; Auer & Jarmai, 2017; Yaghmaei et al., 2019), the SR Thinking Tool focuses on the processual dynamics of research and innovation, with explicit attention to an idealized project lifecycle. In contrast to the RRI tools noted above, and as elaborated in the supplement to this manuscript, the SR Thinking Tool moves away from check-list approaches to responsibility. The Tool encourages double-loop learning (Schön 1983), inviting reflection not only on specific R&I practices but also the broader goals in which R&I practices are situated. Further, RRI keys and conditions invite abstract, general reflection on the goals and values of R&I. In contrast, the SR Thinking Tool scaffolds RRI features among design elements of “entry points” and “guiding questions” linked to real-world examples to concretize and connect RRI concerns to project phases. Finally, also in contrast to the resources reviewed, the SR Thinking Tool does not require background knowledge of RRI (one need not even be familiar with the term or know what it means before using the tool) to be usable and useful.

## Method

**Fig. 1:**
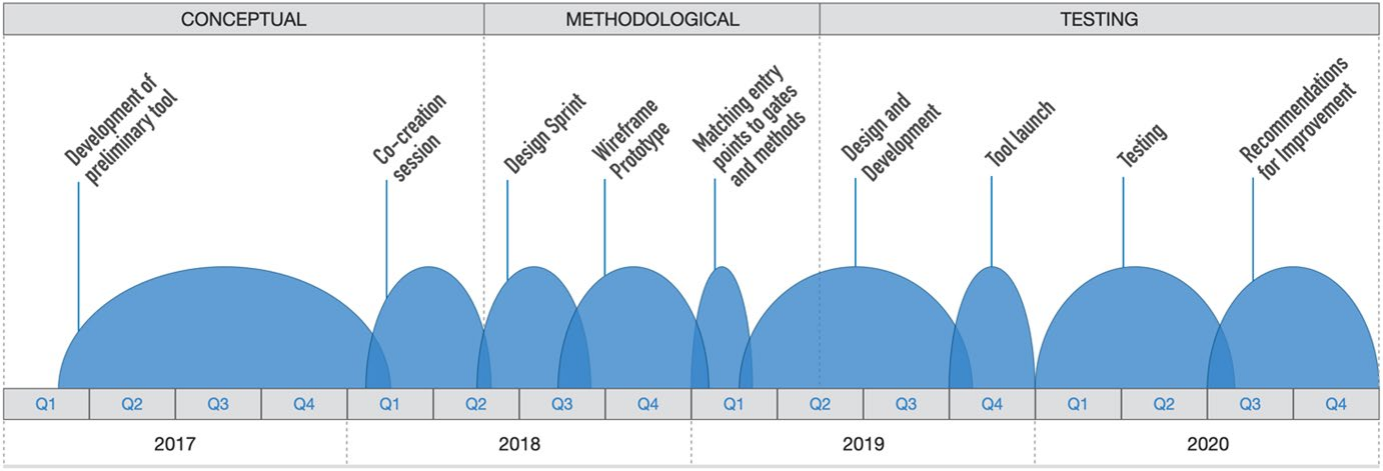
Timeline and overview of the development of SR Thinking Tool across conceptual, methodological, and testing phases

SR Thinking Tool conceptualization, development, and testing unfolded in an iterative and co-creative process. Figure 1 presents a timeline of the activities discussed in subsequent sections. (Additional reporting on in-depth methodological development may be found in the supplementary material to section 2).

### Conceptual Development of the Societal Readiness Thinking Tool

Conceptual development of the SR Tool consisted of a comprehensive literature survey of peer-reviewed scholarly papers using Web of Science, Scopus, and CORDIS (supplement 2.1, Table S1, Figure S1). A total of 1,026 titles and abstracts yielded 171 relevant articles, the contents of which were organized into RRI conditions and keys across research design, data collection, analysis, and dissemination phases and further refined through co-creation sessions with 25 RRI experts from the NewHoRRIzon project. Subsequently, in a two-day Design Sprint (Knapp et al., 2016), the team developed the architecture and web-design concept for the tool.

### Methodological Development of the Societal Readiness Thinking Tool

Materials from the Design Sprint were prototyped by a graphic designer before being built as a web-based wireframe, launched in October 2019, by Computer Science students at Aarhus University. A smaller team of four RRI and research policy experts identified and matched user entry points to the conceptual and processual elements of the SR Tool according to a deliberative, consensus sorting process (Bernstein et al., 2019). Each question was further linked to one of 28 supporting methods, allowing users to seek additional reading and guidance to enable implementation in practice.

### Testing the SR Thinking Tool

Testing of the online version of the SR Thinking Tool proceeded according to Birkving et al. (2019). A full account may be found in the supplementary materials section 2.3. Testing occurred in three phases, covering a range of intended initial user communities: researchers writing funding applications, managing research projects, or conducting research, as well as policy actors and research support staff. Initial testing engaged three subgroups of the NewHoRRIzon project. Subsequently we conducted six focus groups (Wilkinson, 2004) at four Dutch universities (two comprehensive universities and two specialized universities: one, a technical university; the other, an institution focused on social sciences and humanities) and two university medical centres (a total of 38 participants, with 4 to 10 participants per focus group). Finally, six Thinking Aloud interviews were organized, based on Boren and Ramey (2000), with interviewees representing a diversity of academic disciplines (astronomy, environmental sciences, biology, law, psychology, and public health). From all focus group and thinking aloud sessions, notes and results were analysed qualitatively using ATLAS.ti software.

## Results

Below, we present conceptual, methodological, and user-testing results associated with the process of creating the SR Thinking Tool.

### Conceptual Development: Keys and Conditions of RRI in a Stage-Gating Structure

In reviewing the literature, we recognized two distinct approaches to RRI: the academic procedural approach (which has also gained traction in select national-level research and innovation policy bodies) and the more input-focused policy approach (which rose to prominence in the European Commission research and innovation framework Horizon 2020) (Owen & Pansera, 2019; Pellé & Reber, 2015). Each of these approaches offered valuable material for complementing and complicating technology readiness concepts and trajectories with broader societal considerations and ethical concerns.

The procedural approach, originally advanced by Stilgoe et al. (2013), suggests specific ‘dimensions’ of responsible innovation. Our review of more than 200 articles related to RRI illuminated a consistent set four such dimensions: anticipation, reflexivity, inclusion, and responsiveness (See supplementary Table S2) (Burget et al., 2017). In reviewing the literature, we observed ways in which these dimensions were frequently operationalized and envisioned as ‘conditions’ when discussed in more procedurally-oriented efforts to “satisfy” concerns about responsible development of research and innovation (Pellé, 2016; Pellé & Reber, 2015; Thapa et al., 2019). In designing the SR Thinking Tool we favored this terminology of ‘conditions’ because of our intent to offer a procedure-based scaffold (e.g., broken out by idealized stage-gates) for helping researchers and innovators better “satisfy” broader societal and ethical concerns in the course of research and innovation. The term “conditions” invoked for us a notion of the considerations to include in R&I to “satisfy” broader societal and ethical concerns, thus potentially enhancing the “societal readiness” of R&I developments.

Together, these four RRI conditions create a basic framework for helping researchers reflect on intended and possible unintended outcomes and applications of research and innovation in various societal contexts (anticipation). They encourage researchers, innovators, funders, and science-policy makers to raise questions about whose voices and interests should be considered in the design and development process (inclusion); about the underlying goals, motivations, assumptions and worldviews driving the work (reflection); and about how to respond to the knowledge developed through such reflections (responsiveness *qua* double-loop learning) (Foley & Wiek, 2017; Stilgoe et al., 2013). Several national science and technology funding programs adopted the dimensions of anticipation, reflection, inclusion, and responsiveness, including the UK’s Synthetic Biology Roadmap^2^; the framework for Responsible Innovation under BIOTEK2021, IKTPLUSS, NANO2021 and SAMANSVAR programs from the Research Council of Norway^3^; The platform for responsible innovation out of the Netherlands Organization for Scientific Research^4^; and the UK Engineering and Physical Sciences Research Council.^5^

In contrast, the input-based or policy-approach to RRI stands up pillars or “key ingredients” to be considered in the course of R&I. These RRI features were originally promoted in the European Commission’s Eighth Framework Program for research and innovation, Horizon 2020 (EC, 2013) and consists of six distinct keys (public engagement, open access, science education, gender, ethics, and governance)^6^ (EC, 2012) (see supplementary Table S3). The keys consolidate and advance a legacy of accounting for social dimensions of research dating back to Framework Program 2, when the Commission first included ethics as a requirement in research on informatics in medicine (EC, 1988). Subsequently, the Commission added attention to reflecting on environmental impacts in Framework Program 3 (EC, 1990), and to addressing gender inequality beginning in Framework Program 5 (EC, 1999). Framework Program 7 further advanced efforts to enhance societal dialogue with scientific practice (EC, 2006).

**Fig. 2:**
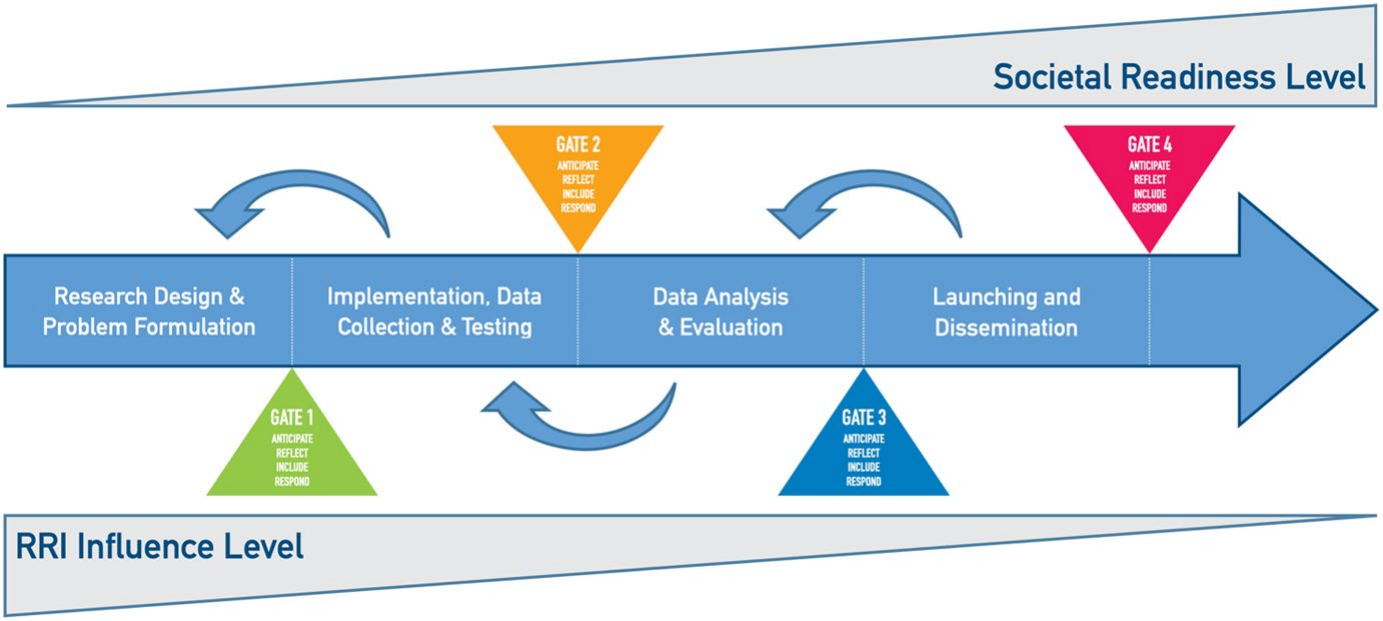
Stage-gate model of Societal Readiness Thinking Tool. Note: Some elements in this figure are inspired by Fig. 9.1 in Lettice et al. (2017)

The SR Thinking Tool pairs and situates conditions and keys in a familiar business-based product development stage-gating concept (Cooper, 1990). In this way, the SR Thinking Tool stage-gating builds upon the procedural dynamics of Stilgoe et al. (2013). Stage-gating divides R&I processes into discrete stages punctuated by decision gates, which may be subject to assessment. Advancing by stage could be made contingent on formal or informal approval (Nathan, 2015; Stilgoe et al., 2013), although this is not how the SR Tool is currently operationalized. Rather, we the apply stage-gate structure to punctuate project lifecycles with recognizable passage points before which select questions of RRI become more or less relevant (Fig. 2).

The SR Thinking Tool differentiates four stages common to research projects. Stage 1 captures ideation processes, where new ideas for discovery are conceptualized, research problems are formulated and appropriate procedures for data collection and experimentation are planned. Stage 2 covers activities related to implementation, data collection, and experimental testing. Stage 3 encompasses data analysis, evaluation, and interpretation of results. Stage 4 covers the launching of project outcomes and the dissemination of results to relevant stakeholders, researchers, and public audiences.

Although described as distinct within a sequence, these stages are not reliably consecutive. We fully acknowledge that stages are discrete and necessarily sequential in time, but the overall progression of R&I may not be linear: at different points of stage-gating, one might step back to re-consider elements previously fixed in earlier stages before proceeding through a subsequent gate, or flag considerations for resolution in future stages (Mulgan, 2006). Research and innovation projects are increasingly organized in large-scale consortiums with multiple, closely connected research agendas, bringing together partners from different countries and contexts. This implies that several interacting research processes may be at play in the same project, which in turn makes a linear, temporal accounting of processes problematic (Felt, 2016). In practice, we therefore interpreted that iterating among phases be a necessary and essential feature enabled by the SR Thinking Tool, without necessarily constraining users to a linear course. For this reason, the eventual web-layout for the tool presented stages along the circumference of a circle.

Drawing on insights from the RRI literature, we developed a set of generic questions for reflection to be addressed at each project stage. These questions were developed to spur general reflexivity about how RRI may be integrated into different stages of early discovery, research, and innovation processes—from the ideation phase, where new ideas for discovery are conceptualized, to the launching of project outcomes, where results are disseminated to relevant stakeholders, researchers, and public audiences.

As demonstrated by the representation of the upper and lower triangles in Fig. 2, significant potential opportunities for cultivating alignment between potential technologies and long-term societal interest occur in early stages of a project (Lettice et al., 2017). Researchers and innovators who invest considerable efforts early in a project may be better positioned to ensure their work develops in a manner responsive to broader societal interests and ethical concerns. In later project stages, when the possible societal impacts become more apparent, it may be increasingly challenging and costly to choose a new course for the project (van de Poel et al., 2017). Resolving this tension of timing requires a well-developed “sociological imagination” (Mills, 1959). It calls for critical and sometimes abstract thinking about complex paths through which planned projects may influence (and be influenced by) society. In acknowledging these challenges and to facilitate such imagination, our SR Thinking Tool poses its largest share of questions in and around stage one.

#### Operationalization: Guiding Questions and Supporting Resources

We operationalize the SR Thinking Tool through generic reflective questions intended to aid identification and accounting for key societal dimensions of innovation at different stages of a project (see Tables 1, 2, 3, 4 for questions offered in respective stages). Many of these questions are adopted and adapted from the literature (Andersen, 2017; Callon et al., 2010; CEN, 2017; Jirotka et al., 2017; Kupper, Klaasen, et al., 2015a; Kupper, Klaassen, et al., 2015b; RRI-Tools, 2018; Stahl et al., 2015; Stilgoe et al., 2013). Each table pertains to one of the four stages bound by the “gates” represented by the triangles in Fig. 2. To proceed from one stage to another, project participants are expected to carefully consider the proposed questions in the associated table and, ideally, modify questions tailored specifically to their own needs. While not all questions are equally relevant to all projects, carefully reflecting on whether a question may be relevant, and why it may *not* be, represents an important intent of the overall exercise.

**Table 1:** Gate 1—Research design and problem formulation

	Anticipate	Reflect	Include	Respond
Public engagement	How will you ensure that you maintain good relations with your stakeholders? At which phases in the project will stakeholder involvement have the most crucial impact, and why?^b^ How early in the project do you plan to involve potential stakeholders?^g^ Who will be the primary users/beneficiaries of the project, and could this change? Who might be excluded from the benefits of the project, and how will you address this? How will different stakeholders benefit from your project?	Have you considered alternative definitions of and approaches to the problem at stake?^c^ Have relevant stakeholders been involved in defining the research problem? Who are the relevant stakeholders of your project?^e^	Which actions will be taken to involve all potentially relevant stakeholders including researchers, representatives from industry, policy-makers and civil-society actors in the project?^h^	Is it possible to change problem formulation or project design in response to changing stakeholder viewpoints or unforeseen ethical issues arising throughout the project?
Open access	Which aspects of the project do you plan to make open access? ^b^ What can you do to ensure that all project partners comply with your open-access strategy? Could pre-registration ensure transparency and openness in this project?	What are the potential barriers to making your data, coding and publications open access and how could these barriers be addressed? Do you have valid reasons for not preregistering you research?	What can be done to make proceedings and the final results of your project easily accessible and intelligible to a diverse set of stakeholders?^d^ With whom do you plan to share the results of your work?^b^	
Science education	Will the project contribute new knowledge of relevance for science education, and how? Could your project benefit from involving citizens in data collection and analysis, and how?	Can RRI perspectives be integrated into the training and supervision of project staff, and how? What would it take to better accommodate citizens interested in contributing to your work, and how? How do you plan to communicate the uncertainty of your research?	Which stakeholders will take part in the project’s education and training activities, and why?^b^ Will your education and communication activities be tailored to specific stakeholder groups, and which?^b^	
Gender	How may your project contribute to improve gender balance in society? Could the outcomes of this project benefit from incorporating a gender dimension into research content, and how?	What are the barriers to gender balance among researchers and leaders in this project and how can these be addressed? What are the possible gender and sex dimensions of the problem at stake?	What can be done to ensure gender balance among researchers and leaders in this project? What can be done to ensure gender diversity among research subjects?^c^	
Ethics	Why should this project be done?^a^ What ethical issues could your project potentially give rise to?^b^ To what extent will you be able to predict the long-term societal outcomes of the project?^a^	Which actions should be taken to ensure research integrity and compliance with ethical standards in the project?^b^ Does your project involve any risks of negative impacts, and which?	Who will be involved in identifying the ethical issues and possible solutions to these issues in your project, and how?^b^ Which actions will be taken to ensure diverse perspectives on the potential ethical issues arising in your project?	

*Sources*: The following questions were adopted or adapted from existing work. ^a^Jirotka et al. (2017), ^b^RRI-Tools (2018), ^c^Kupper, Klaasen, et al. (2015a), ^d^Andersen (2017), ^e^Stahl et al. (2015), ^f^Stilgoe et al. (2013), ^g^Callon et al. (2010), ^h^Kupper, Klaassen, et al. (2015b), CEN (2017)

**Table 2:** Gate 2—Implementation, data collection & testing

	Anticipate	Reflect	Include	Respond
Public engagement	Will any potentially relevant beneficiaries or end-users be missed by the selected method for data collection? How might the project benefit from involving stakeholders in identifying methods for data collection and empirical testing?	Who have been involved in designing the data collection / testing? How has the nature and purpose of the project been communicated to external stakeholders?^f^ Did the data collection give rise to new consideration about potentially relevant stakeholders, and which?	How will you ensure that all stakeholders feel empowered to voice their opinion?^c^ How will you ensure that all relevant stakeholders have the information they need to engage in a meaningful dialogue about proper procedures for data collection and testing?^g^	Is it possible to change procedures for implementation, data collection and testing in response to ethical issues or stakeholder viewpoints in this phase?
Open access	How may the selected methods for data collection and testing best be documented to ensure transparency and allow for replication and knowledge transfer?	How do you plan to document your methods for data collection / testing in an intelligible and transparent way? What are the potential barriers to making documentations of data collection and testing publicly accessible (e.g. intellectual property rights, competing interests)	With whom will you share potential documentations of data collection and testing?^b^	
Science education	Will the project contribute new methods and techniques of relevance for other researchers and practitioners?	Will it be possible for interested citizens to contribute to the collection of data, and how? How can you ensure that interested stakeholders understand the purpose and approaches of the project?	Which stakeholders are taking part in your education activities, and why?^b^ If your project contributes new methods and techniques of relevance for other researchers and practitioners, how do you plan to support the education of these groups?	
Gender	Will the selected methods for data collection / testing, and sample-size allow for nuanced analysis of possible gender- and sex-related differences and similarities?	Have gender and sex related issues been taken into consideration in the selected methods for data collection and testing, and how? What is the sex composition of the subjects included in the collected sample? Will it be possible to change procedures for data collection and testing to allow for nuanced gender and sex analysis?	How do you plan to identify participants that do not identify as men or women (e.g. non-binary or gender fluid subject) in the data collection?	
Ethics	Can you imagine possible scenarios of misuse associated with the methods and data you are using?^i^	Is the planned research methodology ethically acceptable, including aspects related to data collection and data storage?^a^ Does your data collection require informed consent from the participants? Does your project involve any risks of breach of confidentiality and what might they be?	Who have been involved in identifying the ethics-related issues to be considered in the data collection?^b^ Have certain groups of potential participants been excluded from the data collection due to ethical concerns, and how may this limit your analysis?	

*Sources*: The following questions were adopted or adapted from existing work. ^a^Jirotka et al. (2017), ^b^RRI-Tools (2018), ^c^Kupper, Klaasen, et al. (2015a), ^d^Andersen (2017), ^e^Stahl et al. (2015), ^f^Stilgoe et al. (2013), ^g^Callon et al. (2010), ^h^Kupper, Klaassen, et al. (2015b), CEN (2017)

**Table 3:** Gate 3—Data analysis and evaluation

	Anticipate	Reflect	Include	Respond
Public engagement	Which stakeholders will benefit from your result and which will not?	Who have been involved in data-analysis and evaluation, and why? Did the data-analysis and evaluation give rise to new considerations about potentially relevant stakeholders, and which?	How will you ensure that all stakeholders have the information they need to engage in a meaningful dialogue about data analysis and evaluation? Have the results been discussed with different types of stakeholders to allow for alternative interpretations?	Is it possible to change procedures for data analysis and evaluation of project results in response to ethical issues or stakeholder viewpoints in this phase?
Open access	How may the data analysis and evaluation best be documented to ensure transparency and allow for replication and knowledge transfer?	Did you document your data analysis / evaluation in an intelligible and transparent way, and how? What are the potential barriers to making code-scripts and documentation of the full analysis publicly accessible (e.g. intellectual property rights, competing interests, confidentiality etc.)?	With whom will you share the documentation of your analysis and evaluation?^b^	
Science education	Will the project contribute new analytical and evaluative methods of relevance for other researchers and practitioners, and how do you plan to support this? What do people not participating in the project (teachers, students museums, Civil society organizations) need to know about the data analysis and evaluation of project results to learn about/ engage with the outcomes of your work?	How may interested citizens contribute to your data analysis?	What types of training do you provide for citizens to contribute to your data analysis?	
Gender	How may your findings impact gender norms and gender relations in society?	Has your data analysis paid attention to possible gender- and sex-related differences and similarities, and how?	Have you analysed possible interactions between gender and sex and other sociodemographic variables such as class, ethnicity, race, nationality and age, and how?	
Ethics	Can you think about beneficial applications of your results beyond the original scope of your work? Can you imagine possible scenarios of misuse?^i^ Could your findings be misinterpreted, and how?	What ethics-related issues are involved in your data analysis? What types of sensitivity analysis have been used to test the robustness of your methods and results?	Did your analysis devote attention to possible variations across sub-groups of participants, and how?	

*Sources* The following questions were adopted or adapted from existing work: ^a^Jirotka et al. (2017), ^b^RRI-Tools (2018), ^c^Kupper, Klaasen, et al. (2015a), ^d^Andersen (2017), ^e^Stahl et al. (2015), ^f^Stilgoe et al. (2013), ^g^Callon et al. (2010), ^h^Kupper, Klaassen, et al. (2015b), CEN (2017)

**Table 4:** Gate 4—Launching and dissemination

	Anticipate	Reflect	Include	Respond
Public engagement	How can your stakeholder engagement experiences inform future engagement activities in your research area?	“Does your dissemination plan address the relevant users and beneficiaries of the project?	Is your dissemination plan tailored to the needs and characteristics of specific stakeholder groups?^b^	Is it possible to change your launching and dissemination activities in response to needs and concerns of societal actors?
Open access	Who will be responsible for maintenance and storage of the open-access information after the project ends, and for how long? Could the data collected as part of this project be useful for other research purposes, and which?	Is the open access information accompanied by clear and transparent documentation of data editing, statistical procedures and analytical decisions made through-out the project? Is the information made open access accompanied by clear specifications on data structure and variable descriptions to allow for replications or new research purposes?	Will all open access information be available in English? Is licensed software required to benefit from your open access information?	
Science education	How may your results contribute to the public interest in and understanding of science? How may the results of this project be used in the education of future generations of researchers and engineers?	Which other communication channels than peer-reviewed journals will you use to communicate your work ?	Will the results of your project be communicated for science education purposes in other languages than English??	
Gender	What impact do expect your project will have on gender equality?	What is the gender balance among the authors on the peer reviewed papers resulting from this project? Will both women and men be taking roles as leading authors? Are the results reported by sex and gender in your publications, and how? What can be done to help support the future career of both men and women junior scholars in the project?	How will you communicate your results in a way that does not reinforce gender stereotypes?	
Ethics	Can you imagine possible scenarios where the outcomes of the project may be misrepresented or misconstrued in the public discussion?	How will you brief the participating research subjects about the project results? What can be done to ensure that your results are not misrepresented or misinterpreted in the public debate?	Do you plan to involve possible stakeholders in discussions about the ethical implications of your project results?	

*Sources*: The following questions were adopted or adapted from existing work. ^a^Jirotka et al. (2017), ^b^RRI-Tools (2018), ^c^Kupper, Klaasen, et al. (2015a), ^d^Andersen (2017), ^e^Stahl et al. (2015), ^f^Stilgoe et al. (2013), ^g^Callon et al. (2010), ^h^Kupper, Klaassen, et al. (2015b), CEN (2017)

To facilitate the design and pursuit of societally appropriate research and innovation, the SR Thinking Tool offers users introductions to concrete methods and resources for further enhancing responsible consideration of and responsiveness to societal and ethical concerns in project-driven research, along with suggestions for further readings and case-examples of RRI applications. The 28 linked methods and resources included in the SR tool, at the time of this writing, were added based on three considerations. First, they needed to be generic enough to be relevant across a wide range of different research disciplines. Second, the resources needed to provide researchers with concrete methods and tools ready to use in research projects. Third, the methods should be useable to advance broad combination of RRI keys and conditions. The methods and resources are also connected to the sets of questions gathered in the SR tool. For example, when a researcher is presented with a question for reflection, she or he is also presented with relevant methods and resources for addressing said question. One such resource is the Gendered Innovations project (see Table S4), which offers practical methods for gender and sex analysis in science. In the Thinking Tool, the description of Gendered Innovations is linked to reflective questions about gender in knowledge production, e.g. “What are the possible gender and sex dimensions of the problem at stake?” (Table 1) and “Has your data analysis focused attention to possible gender- and sex-related differences and similarities, and how?” (Table 3). If uncertain about the issues in question, users can investigate the proffered methods and resources to build a more complete understanding before answering.

### Online Deployment and Tool Layout

The SR Thinking Tool was designed and deployed for online use (http://thinkingtool.eu/). Our primary initial target group was academics, whose research and innovation trajectories often start with an idea seeking funding. Increasingly, funders expect applicants to address a diverse set of issues such as ethics, gender, and open science. These expectations of applicants are what we leveraged to create “entry points” to the SR tool. By “entry points” we mean instigating reasons motivating a user to seek out SR tool. Answering SR tool questions is intended stimulate thought and subsequent decisions about research and innovation practices. Researchers may use responses to these questions when preparing a particular proposal, crafting protocols, drafting intermediary and final reports for funders, or any number of other moments in project life.

**Fig. 3:**
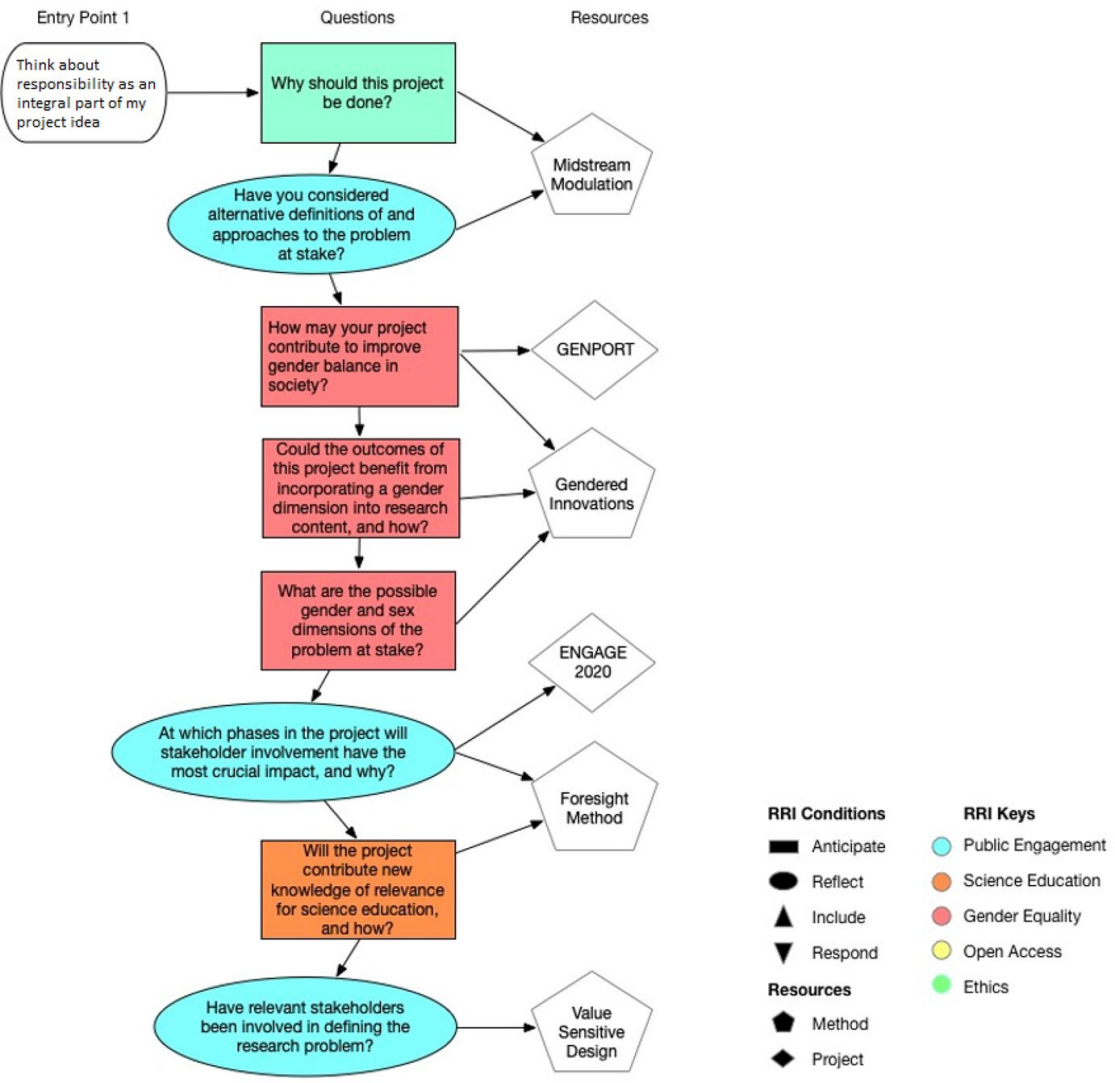
Back-end layout—Gate 1, Entry Point 1. In this example, the user has chosen to, “think about responsibility as an integral part of my project idea” (entry point). She or he has entered the tool at Gate 1 and wants to reflect on questions pertaining to four of the keys (public engagement, science education, gender equality, ethics) and two of the conditions (anticipate, reflect). The different colors and shapes of the questions specify what key and condition each question is tied to. The arrows on the right side of each question point to relevant methods and project resources that may aid in addressing the question

**Fig. 4:**
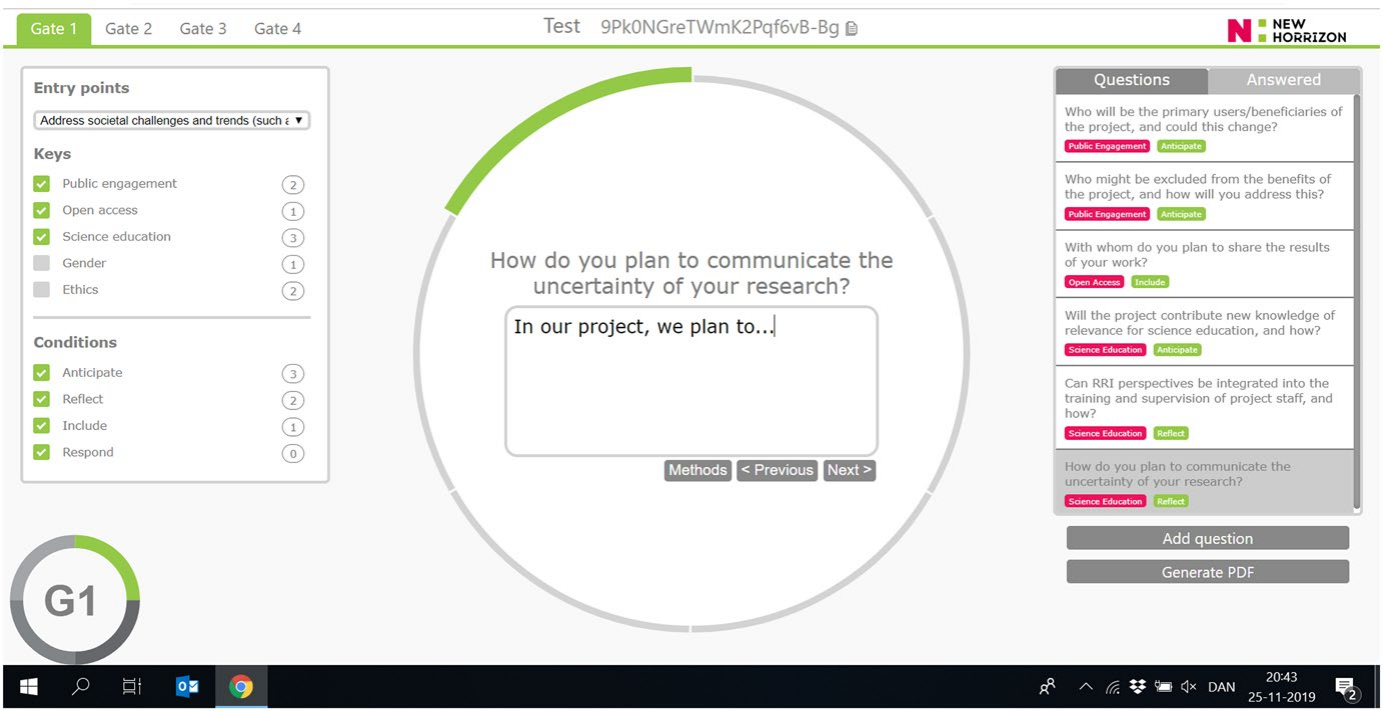
Front-end view of entry points drop down list (right) and selection pane (left) of RRI keys and conditions. In this figure, options selected by a user who wants to “address societal challenges and trends” and wants to reflect on questions pertaining to three selected keys (public engagement, open access and science education) and four conditions (anticipation, reflexivity, inclusion, and responsiveness). In the center of the circle (the non-linear portrayal of stages), the user is offered a number of questions specifically tailored to the selected entry-point, keys, and conditions

When entering the online portal, users^7^ are asked to select the current research phase of their project, as well as choose from among a suite of possibly relevant entry points to start use (see Table S5 for a complete list of entry points). For example, one entry point included in stage two asks whether researchers are “here” to better, “Engage stakeholders in the implementation, data collection, and testing of the project.” Whether they select a stage, entry point, RRI condition or key, the user will be presented with associated questions. This is made possible by the way such content is tagged by relevance to RRI keys and conditions (See Fig. 3 for back-end layout). Content navigation is further augmented by a drop-down list of RRI keys and conditions (see front-end layout, Fig. 4).

Once users select among an initial set of questions, they may enter response text and access guiding methods and resources linked to each question. Users may choose to respond as individuals, or, we hope, take the questions away for use in project-team discussions and deliberation to ‘open-up’ (Stirling, 2008) conversation among peers (i.e., “thinking with” others). As users respond to questions, the circle circumscribing the SR Thinking Tool gains color, with remaining, unanswered sections greyed. The Tool allows users to propose new reflective questions connected to societal readiness to be integrated into the tool, ensuring ongoing user-driven refinement based on respondents’ domain-specific knowledge. Upon completing navigation through the SR tool, users may generate PDF files containing the questions they encountered, their responses, as well as supporting methods and resources of relevance to their research and innovation efforts. All data generated by registered users (who, by registering, obtain ability to “save and continue”) are stored on a secure server at Aarhus University and accessible only by project-specific codes generated on sign-up.

### SR Thinking Tool User Testing Results

Initial SR Thinking Tool testing with user focus groups sought to determine resource efficacy (in supplement 3.3, we present the general feedback received from the “alpha testing” with three NewHoRRIzon international meetings, indicated in section 2.3). We defined efficacy as a function of whether users reported the tool to facilitate thinking about broader societal issues and ethical concerns associated with research and innovation. In addition, we sought feedback on tool usability. Systematic analysis of focus group conversations and Thinking Aloud interviews are presented in combination in related analytical categories.

Each focus group and Thinking Aloud interview lasted for 1.5–2 hours. Participants used the SR tool in real time during focus groups and shared their strategies, behaviours, experiences, and ideas as guided by a set of pre-set questions. Questions covered user’s initial understanding of RRI and their expectations of the resource before use. Users were then invited to open the SR Thinking Tool without introduction (the envisioned situation for new user encounters) and start exploration online. After a general exploration of the tool, each focus group went into greater depth reflecting on one each of the five RRIs keys. The ethics, gender, public engagement, and science education keys were covered by one group each; open access by two groups. Sessions ended by reflecting on how users experienced the SR tool and ways they anticipated future use. Because the research staff guiding the focus groups were selected for their experiences advancing gender, open science, and ethics keys, discussions emphasized keys more than conditions.

In the majority of cases, participants understood the general lay out of the tool according to stages and keys. Conditions and the entry points were less intuitively recognized. Only after explanation of how the sets of questions were designed (based upon the tables combining keys and conditions, e.g., Tables 1–4), did respondents recognize conditions and entry points. Systematic analysis of focus group and Thinking Aloud interview data allowed us to demarcate several types of feedback. We gleaned information about users’ expectations of the SR Thinking Tool, including what they appreciated about the prototype; what seemed unclear about the structure of the SR tool or its content; suggestions for improved text or deployment; and observations related to content and SR tool structure. We gained insight into technical, visual, and substantive aspects of the SR tool, including, for example (below, the “feedback aspect” in text at left, with exemplary feedback in (*italics and parentheses*), at right):

- Technical aspects *(cue: [Researcher] clicks a key, thereby deselects this key, and then deselects all the keys while selected the initially deselected key.)*
  [Researcher, law]
- Visual aspects *(‘The overall look and feel is nice, not a Windows 95 impression.’)*
  [Focus group university medical centre 1]
- Structure *(‘The conditions do not speak to me. Are they essential?’)*
  [Focus group technical university][Focus group technical university]
- Functions *(‘Don’t call it ‘methods’, call it ‘help’.)*
  [Interview, project officer biology]
- Questions *(‘The question about English [whether this is the only language used for communication in the project] is a good one.’)*
  [Focus group comprehensive university 1]
- Effects *(‘It widened my perspective; I now know what the five elements of Responsible Research and Innovation are.’)*
  [Focus group specialized university—social sciences and humanities]
- Contexts of application *(‘I am convinced that they [the questions] would have been useful [when writing the proposal].’)*
  [Interview, astronomer]

User feedback indicated in general that questions included in the tool were widely appreciated. Focus group participants and interviewees consider questions to be relevant, useful, and practical.

Feedback to-date suggests questions stimulate reflection on issues that researchers find themselves confronted with, but not always familiar with or consciously thinking about. We took these results as indication of the prototype’s promise in translating abstract RRI concepts into meaningful question sets to support integration of broader societal issues and ethical concerns into project work. From a more practical perspective users, indicated that when answering questions in detail, it would be helpful if material could be directly used in other contexts, e.g., fed into grant application forms, ethics application documents, or reports to funders.

User feedback generated helpful findings for further development of the SR Thinking Tool. Still, a range of structural features and functions of the SR tool were not immediately evident to a significant share of users. For example, the label ‘gate’ confused users unfamiliar with the concept (this group mentioned thinking in terms of ‘phases’ rather than ‘gates’); and the ‘methods’ button proved difficult to find. A first impression of the SR tool, shared by many, was of being overwhelmed by the number of implicit considerations made explicit. For example, ‘*It is not intuitive, many researchers will not read it [the introductory text] and will just start using it [the tool]*’ (Focus group comprehensive university 1) and ‘*Make it less complex and more intuitive*’ (Focus group technical university). Such feedback indicated to us a need to further refine presentation and delivery of the SR Thinking Tool to make it as intuitive and accessible as possible.

### From Opportunistic to Strategic Uptake of the SR Thinking Tool

As of 10 July 2021, 700 user projects have been registered online. Excluding “test” entries (so named), approximately 200 of these projects seem to be actively using the SR Tool across one or more stages; on average each of these users engage with six questions. Such users may have been drawn to the tool by the broader dissemination efforts of the NewHoRRIzon project, which supported development of the SR Thinking Tool. Since launch, we have increasingly moved toward more strategic and systematic approaches to identifying and connecting with researchers and research managers and administrators, and funders.

At present, until additional funding can be accessed to support further development, strategic opportunities remain connected to author networks. For example, Erasmus University Rotterdam is now set to include the SR tool in their institutional ‘Evaluating Societal Impact’ strategic project to support researchers when considering possible societal impacts of their work. Separately, discussions with the Brazilian Agency for Support and Evaluation of Graduate Education (CAPES) and the Brazilian Forum of Pro-Rectors for Research and Graduate Education (FOPROP) have generated interest in the potential for the SR tool to be adapted as a component of the self-evaluation protocols in the country. In the context of European funded research, the resource itself has been mentioned as a product in Horizon Europe topics (e.g., HORIZON-CL3-2021-SSRI-01–05). A full elaboration of a strategic approach to deployment and integration of the current version the Societal Readiness Thinking Tool among key research management, administration, and funding audiences is beyond the scope of this article, but our initial successes with Erasmus, CAPES, FOPROP, and Horizon Europe suggest a range of worthwhile avenues to explore.

## Future Work and Discussion

We focused our first iteration of the SR tool on project-based academic research, while envisioning ample possibilities to create tailored versions of the tool for an array of additional audiences (e.g., research funders, research managers and administrators, businesspersons, etc.).^8^ Taking initial user tests into account, we have adopted several next steps for development. First, we will optimize presentation of questions. This will include shortening and clarifying the introduction (for example to specify intended value-added and prospective users). Second, and related to the use-case tested in this article, we will enhance visual and technical elements. A prime example here being increased ease of discovery and access to the ‘methods’ function.

More broadly, we will enhance the design to enable application in a wider range of use contexts. Iterations of the Societal Readiness Thinking Tool may be tailored for research managers, members of a project advisory boards, business stakeholders, non-governmental partners, or research funders who wish to encourage more attention to the societal dimensions of R&I at policy-, program-, or project levels. To do so, additional critical stage-gates could be added, or current gates modified; for example, we imagine an additional stage-gate related to “agenda setting” could be of use to funders. Relatedly, the tool could be improved by developing additional entry points and probing questions specifically tailored to new user groups. For instance, with minimal modifications, the SR tool could be used to teach students about ethical or gender issues in educational settings. To take another example, enabling access for public commentary or engagement could be useful for validating or field-testing assumptions about social dimensions of research and innovation by teams or organizations seeking to reach toward improved societal readiness.

One application of greatest priority will be in contexts where research funders or business investors deploy the tool to enhance societal readiness across a range of projects. We consider funders and investors as key user groups given their role in establishing integrated research networks capable of conducting societally responsive science and innovation policy (Braun, 2003; Klerkx & Leeuwis, 2008). For example, some EUR 28-billion of EC H2020 funding programs and call topics were tailored to address societal challenges, as defined in Europe's 2020 strategy for smart, sustainable and inclusive growth. An additional EUR 18-billion were devoted to industrial leadership initiatives, as well as seeding projects designed to enhance participation and societal responsiveness of research infrastructure across Europe. Historically, research agendas and objectives structuring framework programs are predefined by the Commission in interplay with the European Parliament, the Member States of the European Union, and scientific and business advisory bodies, with rather limited input from broader societal actors and publics (this despite such publics, when supported as in the CIMULACT project, often provide qualitatively different and useful inputs representative of societal interests (Rosa et al., 2018)). A structured resource like the SR Thinking Tool may be used to make future discussions on research prioritizations more responsive to the full gamut of societal values, needs, and expectations.

The SR tool’s attempt to support the integration of insights and perspectives from broader and more diverse stakeholder-, public-, and epistemic communities distinguishes it from non-RRI-based resources, like sustainability-oriented innovation (SOI) (Buhl et al., 2019). The SR Tool and SOI share a common interest in integrating divergent thinking with traditional approaches to technology development, to support problem reframing, focus on people and lived experiences, and connect to normative guides beyond economic motivations. However, whereas design-thinking traditions have been criticized for being too conservative or preserving the status quo (Iskander 2018), RRI emerges from a scholarly community explicitly grappling with novel and emerging technologies and seeking to explore alternative frames, scopes, and possibilities for these areas of research to contribute to society (c.f., Barben et al., 2007; Robinson, 2009; Aicardi et al., 2018).

Finally, beyond development and strategic application considerations, in-depth evaluation of the effectiveness and impacts of various use cases of the SR Thinking Tool offers another area of future research. With additional funding, qualitative use-case analyses could illuminate impacts of SR TT adoption on, for example, capabilities conducive to supporting scientist and engineers in anticipating and reflecting on social and ethical dimensions of research and innovation processes (O’Donovan et al., 2021). Such studies could provide critical feedback to funding organizations about ways to reconfigure institutional landscapes to be more conducive to anticipatory, reflective knowledge production (Smith et al., 2021). Exploring such use-cases over longer periods of time and across disciplines and sectors could offer robust lessons not only regarding capabilities and institutional forms supportive of integrating social and ethical dimensions of research and innovation, but also tracing longer-lived impacts of such anticipatory and reflective efforts across research practice, business, and policy activities.

## Conclusion

We have outlined the need for and described the development of a Societal Readiness Thinking Tool. The key purpose of the resource is to support researchers and innovators in thinking about ways to align their work with broader societal issues and ethical concerns. Emphasis on “thinking” connotes the centrality of this iterative activity to all phases of research and innovation projects in the making. The SR Tool addresses a recognized gap in the availability of structured, concrete guidance for integrating societal and ethical considerations along multiple phases of research and innovation projects (c.f., Inigo & Blok, 2019; Stahl et al., 2015, 2019). User feedback collected in participant focus groups and user interviews indicates prompting questions and resources are appreciated and deemed relevant, useful, and practical.

Technology Readiness tools often advance technology development independent of broad-based or long-term issues of societal concern. The Societal Readiness Thinking Tool seeks to complement existing TR approaches by gathering, organizing, connecting, and presenting a diverse array of guiding questions related to critical societal dimensions of research and innovation. By disaggregating keys and conditions related to RRI, we hope the tool maintains relevance, noting that concerns of ethics, gender, open access, and stakeholder engagement remain very much alive in Horizon Europe, the now-active ninth European framework programme. Our tool provides an interactive, accessible, and structured way for researchers and innovators to integrate broader societal concerns consistently across stages of innovation, representing a key step for translating good intentions for responsible research and innovation into action.

## Supplementary Information

Below is the link to the electronic supplementary material.

Supplementary file1 (DOCX 80 kb)

## Footnotes

1 Note that all elements of the work (including the literature review, conceptual development, design processes, and user testing) leading to the current iteration of the web-based version of the SR Thinking Tool have been reported in deliverables from the NewHoRRIzon (http://www.newhorrizon.eu) project (cf. Birkving et al., 2019; Chan et al., 2019; Nielsen et al., 2017). We have reused major pieces from these reports (particularly Nielsen et al., 2017) for the purposes of this article, which is the first presentation of the SR thinking Tool in an academic outlet.

2 Available at: http://webarchive.nationalarchives.gov.uk/20130302042701/http://www.innovateuk.org/_assets/tsb_syntheticbiologyroadmap.pdf.

3 Available at: https://www.forskningsradet.no/prognett-biotek2021/Documents/1253970728136 .

4 Largely focused on ethics and integrity. Available at: https://www.nwo.nl/en/research-and-results/programmes/responsible+innovation.

5 Available at: https://epsrc.ukri.org/research/framework/.

6 The omission of governance from the Thinking Tool reflects a recognition of how changes to rules, norms, or practices of research and innovation, e.g., to advance the five keys, necessarily entails changes in governance.

7 The tool may be used with or without registration. Users who register can retain their responses in the tool’s database and return to continue reflection, for example after entering a new phase in the project lifecycle, or after they have discussed answers or shared resources and garnered feedback from project peers and partners.

8 Given limited initial resources, the prominence of researchers and research funders in public R&I activities, and the familiarity of the research team with academic audiences, our first iteration of the SR Thinking Tool focused on non-commercial R&I, often early stage. However, given the importance of commercial sectors in R&I and the share of responsibility necessarily vital in such sectors, we intend to return to this audience in future iterations of this work.
