# Supplementary material for: The Societal Readiness Thinking Tool: A Practical Resource for Maturing the Societal Readiness of Research Projects
Source: Sci Eng Ethics. 2022 Jan 27;28(1):6. doi: 10.1007/s11948-021-00360-3 (PMC8794941; doi:10.1007/s11948-021-00360-3)
Supplement: Supplementary file 1 — Supplementary file1 (DOCX 80 kb) [file 11948_2021_360_MOESM1_ESM.docx]

# SUPPLEMENTARY MATERIAL

# The Societal Readiness Thinking Tool: A Practical Resource for Maturing the Societal Readiness of Research Projects

In this supplement we present additional results from the literature review and development process of the Societal Readiness Thinking Tool.

**Supplement to section 1**

***Supplement 1.3: Brief Review of existing RRI Tools and related resources***

Felt et al. (2018) developed IMAGINE RRI: a card-based method that allows life-science researchers to reflect upon notions of responsibility in relation to their practice, institutional constraints and roles as researchers. This method represents an innovative and accessible resource for stimulating shared reflection about RRI in life-science contexts. However, it does not cover the conditions and keys presented above, and it places less emphasis on the processual dynamics of RRI-related project work than the thinking tool proposed below.

The EU-funded project ‘RRI-TOOLS’ represents another effort to advance adoption of responsibility in research and innovation. In addition to collecting hundreds of tools and resources, the RRI-TOOLS’ “Self-Reflection Tool” guides users in examining RRI-related dimensions in their own projects based on reflective questions tailored to each of the RRI keys as well as for specific stakeholder groups (e.g., researchers, business persons, policy-makers, etc.). The resource, however, does not make accessible RRI dimensions in relation to research and innovation project life-cycles.

Research projects, including ORBIT, COMPASS, and PRISMA, have developed RRI tools for business-specific use (see e.g. Blok and Lemmens 2015; Van de Poel et al. 2017; Auer and Jarmai 2017; Yaghmaei et al. 2019). The core objective of the UK-funded ORBIT project is to “provide services to promote RRI across the UK ICT research community”. The project includes a survey-based self-assessment tool that allows researchers to quantify various dimensions of responsibility in their own R&D activities. Similarly, the EU-funded project COMPASS offers an RRI-related ‘Self-check Tool’ for SMEs in emerging technology industries. The tool asks SMEs to rate their level of responsibility in the following core areas: company management, idea generation and research, development and testing, and market and impact. In a similar vein, the EU-funded project PRISMA developed a tool to assess RRI performance against RRI principles and organizational criteria by piloting the implementation of RRI within eight companies from small to large. The thinking tool we propose differs from these projects’ methods in that we place less emphasis on quantification and more emphasis on spurring higher-levels of reflexivity (c.f., Schön 1980) around responsibility through a stage-gating inspired approach.

Inigo and Blok (2019) attempted to integrate RRI insights with circular economy developments “to address socio-economic and socio-ethical issues,” (p. 286) associated with accounting for long-term undesirable consequences of scientific or technological development (e.g., related to waste generation) or including broader sets of participants. While Inigo and Blok (2019) helpfully point to general classes of activities, like codes of conduct, involvement of ethicists, or performance of technology assessments, the authors do not integrate this information in a way broadly accessible to researchers and innovators seeking assistance across the phases of their project work.

Sjöö and Frishamar (2019) proposed a model for navigating challenges in advancing to demonstration levels of projects. The authors include reference to Technology Readiness Levels as part of a suite of checkpoints related to project feasibility and long-term prospects. Several of the project-internal challenges Sjöö and Frishamar (2019) referenced relate to identifying, enrolling, and mobilizing broad sets of participants to advance project development. One of the advantages of the SR Thinking tool, for this purpose, is the way in which, from earliest stages of scientific or technological project ideation, reflective questions and guidance are offered to bring such participants into R&I processes from the beginning. For example, Gate 1 questions on problem definition include, “Who are the relevant stakeholders of your project?” or “Who will be involved in identifying the ethical issues and possible solutions to these issues in your project, and how?” (see Table 1 for additional examples). Attention to societal concerns in such a manner may contribute not only to improved project development by “balancing the needs, wants, and requirements of different stakeholder” (Sjöö and Frishamar 2019, p. 339) but also to the larger altruistic ambitions of researchers and innovators of “doing good for humanity” (Stahl 2019, p. 8).

Buhl et al (2019) presented an integration of design thinking into sustainability-oriented innovation. The authors emphasize, in a manner well attuned to the aims of societal readiness, that sustainability “is not anticipated as a definite result but rather as a (normative) direction” (p. 1249). Buhl et al (2019) highlighted four particular challenges associated with sustainability-oriented innovation: “innovation scope, user needs and behaviors, stakeholder involvement and assurance of positive sustainability effects” (p. 1250). The authors then argued that design thinking principles related to problem framing, user focus, diversity, visualization, experimentation and innovation offer a way to supplement processes to support the normative direction of sustainability.

**Supplements to section 2**

***Supplement 2.1: Conceptual development of the Societal Readiness Thinking Tool***

The literature guiding the initial development of the thinking tool and the description of existing RRI-related methods and resources was identified through a comprehensive search in Web of Science, Scopus and CORDIS. The literature survey of peer-reviewed scholarly papers was carried out using abstract and title-focused searches in Web of Science and Scopus. We searched for articles, books and book-chapters in English published since 2000, using a broad variety of search terms (Table S1). We read through 1,026 titles and abstracts, ending up with 171 relevant articles (Figure S1). All articles deemed relevant met at least one of the following selection criteria:

1. Articles should add to the conceptual clarification of what RRI is
2. Articles should provide practical ideas or methods on how to mature the societal readiness of R&I projects
3. Articles should offer practical case-examples on how to ensure the social appropriateness of R&I activities.

Table S1: Search terms for Web of Science (WoS), SCOPUS, and CORDIS

| Search string WoS | (TS= (innovation OR "research and innovation" OR technology) AND TI= ("responsible research" OR "responsible innovation*" OR "Innovation trajector*" OR "responsible research and innovation*" OR "broader impacts criterion" OR "value sensitive design*" OR "value-sensitive design*" OR "technology acceptance" OR "social-technological alignment*" OR "social technological alignment" OR "ethical impact assessment*" OR "ethics assessment" OR “human readiness level*" OR "humanreadiness level*" OR "social implications of technolog*" OR "human implications of technolog*" OR "stage-gate system" OR "upstream engagement" OR "system readiness" OR "human factors measur*" OR "human-factors measur*" OR "decision gate proces*" OR RRI OR "human factors readiness level*" OR "human-factor readiness level")) *AND* **LANGUAGE:** (English) *AND* **DOCUMENT TYPES:** (Article OR Book OR Book Chapter)  **Timespan:** 2000-2018. **Indexes:** SCI-EXPANDED, SSCI, A&HCI, CPCI-SSH, ESCI. |
| --- | --- |
| Search string SCOPUS | TITLE-ABS ( innovation OR "research and innovation" OR technology ) AND TITLE ( "responsible research" OR "responsible innovation*" OR "Innovation trajector*" OR "responsible research and innovation*" OR "broader impacts criterion" OR "value sensitive design*" OR "valuesensitive design*" OR "technology acceptance" OR "Social-technological alignment*" OR "social technological alignment" OR "ethical impact assessment*" OR "ethics assessment" OR "human readiness level*" OR "human-readiness level*" OR "social implications of technolog*" OR "human implications of technolog*" OR "stage-gate system" OR "upstream engagement" OR "system readiness" OR "human factors measur*" OR "human-factors measur*" OR "decision gate proces*" OR rri OR "human factors readiness level*" OR "human-factor readiness level" ) |
| Search strategy in CORDIS | We searched for all funded projects mentioning “RRI” or “Responsible Research and Innovation” in the CORDIS abstract database. One-hundred and two FP6, FP7 and FP8 projects were identified using this search strategy. Of these, 23 were considered relevant to the Thinking Tool. The plan is to briefly describe the focus and activities of each of these projects in the “updated” second version of the “Thinking Tool”. |

Articles, books and book chapters

published in English since 2000

:

N: 569 (WoS) + N: 973 (Scopus

(

):

N= 1,542

Reference duplicates

N= 516

Unique articles, books and book

chapters from WoS and Scopus

N = 1,026

References did not meet

criteria A, B or C

N= 855

Articles, books and book chap-

ters informing review

N = 171

Figure S1: Flow diagram of article inclusion and exclusion

A preliminary tabulated version of the Thinking Tool – a proposed set of questions pertaining to a given RRI condition and ingredient at each of the four research phases: research design, data collection, analysis, and dissemination – was developed. The concept and content of the tool were reviewed by some 25 RRI-experts from the NewHoRRIzon project in a co-creation session. This session gave rise to an explicit call for a more user-friendly and online version of the Thinking Tool. The tool creator and eight RRI experts from various research organizations in the Netherlands, Denmark, Finland and Norway participated in a two-day Design Sprint (Knapp, Zeratsky & Kowitz, 2016) to come up with a web-based design for the tool. Design Sprint is a methodological process that emerged from the principles of design thinking. It was selected after reviewing a plethora of user-centered, design thinking, and participatory design approaches (Sanders, 2002) as it enables the team of experts to prototype quickly, include multiple perspectives, and validate the prototype through user testing.

***Supplement 2.2 Methodological development of the Societal Readiness thinking Tool***

The sketches of the Design Sprint were sent to a graphic designer to develop a wireframe prototype for the web-based version of the tool. The wireframe prototype went through two rounds of revisions with the experts. In the web-based version, users are asked to select the current research phase of their project, as well as a relevant “entry point” for using the tool. Specifying entry-points helps users quickly arrive to questions supporting their motivation for using the tool.

Upon finalization of the wireframe design, four experts participated in an iterative process to match specific user entry point to the questions for each key and condition and the research phases, which we termed gates. Each question was further linked with specific R&I methods and research projects with best practices. The SR Thinking Tool currently offers 28 methods, which contains further reading to answer the questions as well as selected EC-funded projects with the purpose of inspiring and informing researchers of RRI best practices.

The web version of the tool incorporated all design elements specified in the wireframe prototype and was developed by Computer Science students at Aarhus University. The web-based version of the SR Thinking Tool was launched in October 2019.

***Supplement 2.3 Testing of the SR Thinking Tool***

AUTHOR offered a full description of the procedure that was employed for initial testing of the online version among NewHoRRIzon participants and implications for the thinking tool. We next tested the SR Thinking Tool in a first round of interactions using three different yet complimentary methods, all involving representatives of the intended user communities. The first method is a demonstration for social labs (SL) during one of their assemblies. SL7 Health, Demographic Change and Wellbeing; SL14 Spreading Excellence and Widening Participation; and SL15 Science with and for Society, consist of researchers funded through H2020, policy makers, funders, and other stakeholders from civil society and innovation organizations. The second method is focus groups with advisors on European grants and the RRI key topics in Dutch universities, because they are an important intermediary between policy and academic practice. We organized a total of six focus groups (Wilkinson 2004) at four Dutch universities (two comprehensive universities and two specialized universities: one, a technical university; the other, an institution focused on social sciences and humanities) and two university medical centres.

Focus group participants included advisers on European grants with expertise in one or more of the RRI keys, as well as staff working at diversity offices and university libraries. We selected such advisers for two reasons. First, they represented a potential user group, as they are involved in supporting applications for funding and in organizing research groups and consortia. Second, due to their professional role, we expected them to be well-aware of the policy context around RRI, current societal readiness related knowledge levels and questions in academic practice. Hence, we expected them to be able to reflect on the efficacy and user friendliness of the tool from their own perspective and the perspective of the researchers that they interact with on a daily basis. In total, 38 advisers participated in the focus groups, ranging from 4 to 10 participants per focus group.

We organized six Thinking Aloud interviews based on method as described by Boren and Ramey (2000). We selected five researchers and one project officer each involved variously in writing funding applications, managing research projects, or conducting research. All interviewees were working at the same single Dutch university or the affiliated university medical centre. The six interviewees represented a large variety of academic disciplines: astronomy, environmental sciences, biology, law, psychology and public health.

While using the tool, interviewees were stimulated to think out-loud during to answer questions about their experiences navigating options or answering questions. Interviewers noted verbal responses as well as non-verbal cues, for example sighing, or waiting, while interviewees used the Tool. Each interviewee focused on one or two RRI keys they deemed most relevant for their own project. Before they used the tool, we asked questions to assess their prior knowledge of and perspectives on RRI. After the interviews we asked questions about their experience in using the tool.

From all focus group and thinking aloud sessions notes were taken and results recorded, which were analysed qualitatively using ATLAS.ti.

**Supplements to section 3**

Table S2: RRI conditions, after Owen et al. 2012; Stilgoe et al. 2013; Foley and Wiek 2017; RRI Tools (2014).

| **RRI Condition** | **Description** |
| --- | --- |
| **Anticipation** | carefully examining both the intended and possible unintended consequences arising from research and innovation activities, including environmental, health-related, economic and social impacts. Anticipatory processes prompt “what if…?” questions that allow researchers and innovators to prepare for and respond to the various uncertainties and dilemmas built into their work |
| **Reflection** | reflecting on the underlying motivations, assumptions and commitments driving research and innovation. It commits researchers and innovators to inquire and challenge the taken-for-granted assumptions structuring their work and makes them attentive to alternative ways of framing the value and societal impact of their ideas, methods and proposed solutions. |
| **Inclusion** | closely related to public engagement and stakeholder involvement. It is about involving relevant societal actors in research and innovation activities from an early stage, and ensuring continuous, open dialogue about desirable and undesirable outcomes throughout the project. Inclusion serves to broaden the ideas, perspectives and worldviews guiding research and innovation activities. |
| **Responsiveness** | aligning research and innovation activities with the new perspectives, insights and values emerging through anticipatory, reflexive and inclusion-based RRI processes. Responsiveness presupposes a will to learn from practical experience and a capacity to translate this learning into better, more responsible research and innovation solutions. |

Table S3: RRI keys, after Geoghegan-Quinn (2012), EC (2016).

| **RRI Key** | **Description** |
| --- | --- |
| **Public engagement** | engaging a broad range of societal actors in the research and innovation process, including researchers, industry, policymakers and civil society actors. |
| **Open access** | making research and innovation activities more transparent and easily accessible to the public, e.g., through open data and free access to publications. |
| **Science education** | increasing society’s general science literacy, e.g., by boosting children’s interest in science and technology, and by equipping civil society actors with the necessary skills to more actively take part in the research and innovation process. |
| **Gender** | promoting women’s participation as researchers and integrating a gender dimension into research and innovation content. |
| **Ethics** | fostering research and innovation activities of high societal relevance, that comply to the highest ethical standards. |

Table S4: Example of methods introduction – Gendered Innovations

| **Project description** | The ‘Gendered innovations’ project develops practical methods for gender and sex analysis in science, engineering, medical and environmental research. The project’s website offers useful conceptual and methodological resources that allow researchers to integrate gender and sex perspectives in their own projects. In addition, the website presents a list of case studies that illustrate how existing projects have benefitted from integrating gender and sex dimensions into research design. |
| --- | --- |
| **Case example** | Historically, crash test dummies have been designed to model the average male body. As a result, the security features of many cars provide less-than-optimal security for pregnant women. For example, in case of a car crash, the 3-point seatbelt increases the force transmission to the abdomen, thus heightening the risk of fetal injury. The integration of sex analysis has led to the development of pregnant crash test dummies and computer simulations that provide better car safety features for pregnant women. |
| **Further reading** | <http://genderedinnovations.stanford.edu/> |
| **Associated key** | Gender |
| **Associated condition** | Reflexivity |

Table S5: Overview of possible entry points at each gate

| Gate 1 | Gate 2 | Gate 3 | Gate 4 |
| --- | --- | --- | --- |
| Think about responsibility as an integral part of my project idea | Engage stakeholders in the implementation, data collection and testing of the project | Address / develop communication around uncertainties related to the output of my research | Reflect on what worked, why; what did not work, why not—with regard to project implementation and findings |
| Identify partners across disciplines and sectors | Communicate with the advisory group on the progress of the project regarding societal challenges and trends | Involve stakeholders and public in considering the validity, reliability and relevance of results | Communicate and disseminate results for maximizing desirable impacts |
| Develop a responsible project implementation plan | Develop ethics, dissemination & communication, gender, data management, outreach and engagement plans | Rethink the analysis and project evaluation in the light of RRI | Spark public discussion about my results through dissemination and communication |
| Address societal challenges and trends (such as RRI & SDGs) | Rethink the project plan in light of RRI | Ensure transparency in relation to data analysis and project evaluation | Think about follow-up projects related to societal challenges and trends |

***Supplement 3.3 SR Thinking Tool User Testing Results***

The three international meetings (section 2.3) with the NewHoRRIzon community included a wider audience in the meetings such as researchers, funders, policy makers, civil society organisations and other sectoral experts. Their feedback to the presentation of the tool confirmed the analysis and further included positive comments and questions, such as:

Three positive comments emerged from the test of the tool

- There was general recognition of the quality and validity of the questions. These refer to issues that are often overlooked, or difficult to attend to by researchers.
- The policy of RRI is largely unknown by researchers or research support staff. However, the keys and conditions are generally recognized.
- An online tool can be a valuable resource in raising awareness of a policy concept, as well as the underlying notions, among academics, and may be useful for education purposes as well.

Open questions remaining relate to:

- User groups with a more applied approach to research have expected the tool to provide quantitative outputs, such as the assessment of societal readiness in the form of a grade or rating.
- How can the tool contribute to preparation of proposal texts? The usability would increase if considerations elicited by the tool’s questions could be directly included in proposal preparation.
- Even though the Tool can be used to guide group discussions and brainstorming sessions, would it be useful to evolve it to allow multi-user interaction with any given project?
